# WHO BECAME CLOSER TO FAMILY AFTER THE PANDEMIC?: THE ROLES OF OPTIMISM AND SOCIAL EXCHANGES

**DOI:** 10.1093/geroni/igad104.0239

**Published:** 2023-12-21

**Authors:** Katherine Fiori, Amy Rauer, Christina Marini

**Affiliations:** Adelphi University, Garden City, New York, United States; University of Tennessee, Knoxville, Tennessee, United States; Adelphi University, Garden City, New York, United States

## Abstract

Dispositional optimism has been associated with healthy aging, as well as coping with and making meaning from life challenges. Social support plays a similarly positive role in promoting adaptation in late life and helping individuals feel closer to loved ones. During the pandemic, disruptions in older adults’ social interactions placed a high demand on both of these potential resources, challenging individuals’ ability to derive meaning from these events. Exploring data from a community sample of 136 older adults (M age = 68, range 50-91; 69% females; 93% White) assessed three times over six months during 2021-2022, we examined how optimism and positive and negative social exchanges predicted changes in attitudes about how COVID-19 shaped family life. We found that T1 optimism predicted changes in older adults’ feeling closer to their families and appreciating them more at T2 (3 months later), above and beyond T1 positive social exchanges, which were only marginally associated. Older adults who were more optimistic at T1 also reported greater declines in tension and strain in their family ties due to the pandemic at T2 - but not at T3 (6 months later). However, T2 negative social exchanges did predict increased reports of family tension and strain from T2 to T3. Findings suggest that as new COVID-19 variants emerged, benefits of optimism waned, but costs of negative social exchanges increased. These findings illustrate how personality, micro-level context (i.e., social interactions), and macro-level context may work together to shape older adults’ views of their families during the pandemic.

